# Nephroptosis

**Published:** 1907-06

**Authors:** R. R. Kime

**Affiliations:** Atlanta, Ga.


### NEPHROPTOSIS.*

**R. R. Kime, M.D.**

The writer advised dividing movable kidney into 3 degrees for convenience in diagnosis.

1st. Where lower half of kidney can be palpated.

2nd. Where whole of kidney can be palpated and fingers carried above upper pole.

3d. Where kidney is freely movable in abdominal cavity.

He referred specially to the nephro-colic ligament of hong year as a factor in these cases and to be used in the operation. It being attached to kidney capsule and colon—tends to pull down on kidney when colon is prolapsed. While the physiological action of colon tended to increase the nephroptosis also to pull on the duodenum which is practically fixed.

This action prevents stomach completely emptying its contests

*Author's abstract of paper read before Fulton Co. Med. Socy.

hence stagnation, decomposition of food and dilation of stomach. He referred to splanchnoptosis frequently accompanying the nephroptosis and must be considered before operating. Advised discrimination and judgment in a differential diagnosis.

Urged the necessity of looking carefully for disease of appendix pelvic and abdominal organs and correcting same at the time of operation.

The kidney is frequently diseased or perverted in its action due to its movability and repeated microscopic examinations of urine are positively necessary for a correct diagnosis. Kidney pads, as a rule, were condemned as doing harm, but general abdominal support of abdominal organs advised. Such supports should reach from pubes to near ensiform cartledge, put on before patient gets up of morning while patient is in reclining position.

Advises decapsulation and anchoring in all cases of nephroptosis where kidney is diseased.

He utilizes the deflected capsule on upper 2-3 of kidney, the nephro-colic ligament and U shaped stitches in lower undenuded third of kidney. Uses a strip of gauze under lower pole for drainage, to increase adhesions and obliterate supphronic space.

Uses only plain sterile catgut numbers 2 and 3 throughout operation—discarded chronic catgut because of irritation and interferring with union. He urged the special care in diagnosis and correction of other abnormal conditions at the same operation and reported cases illustrating this feature and also the benefit of decapsulation when he kidney is diseased and movable.

He called special attention to the nephroptosis build of slender spare-built females that are specially prone to movable kidney, prolapsed colon, involvement of appendix and want of development of pelvic organs. Dr. Kime condemned as next to criminal the removal of ovaries in these cases and advised conservative work and after treatment to stimulate proper development of pelvic organs for future physiological functions.

Dr. E. C. Davis complimented Dr. Kime's work along this line and thought we often made a mistake in disregarding splanchnoptosis when we find nephroptosis with a consequent bad result.

Dr. Kime (closing) said the dropping down of the right kidney and colon may pull on the duodenum and cause much trouble and should be carefully considered in our treatment.

---

The Texas Sanitarium for Tuberculosis, located at Llano, Texas, announces that Dr. T. O. Maxwell, formerly superintendent of the Southwestern Insane Asylum at San Antonio, Texas, and for sixteen years first assistant physician to the State Insane Asylum at Austin, Texas, has purchased an interest in the Texas Sanitarium, Llano, Texas. He is now a member of the board of directors and has been elected Medical Director of that institution and has taken active charge of same. Dr. Maxwell is an experienced institutional man, and devoting his entire time and giving his personal attention to the successful management of this place insures its success beyond a doubt.

---

The medical man who is too busy to read, supplies lots of post-mortem material for men of more studious minds.—Chicago Clinic.

